# Observation of Edge‐Confined Acoustic Hyperbolic Polaritons in van der Waals Materials

**DOI:** 10.1002/advs.202520556

**Published:** 2026-01-04

**Authors:** Tianning Zhang, Xiaojie Jiang, Xiaosheng Yang, Xinliang Zhang, Peining Li

**Affiliations:** ^1^ Wuhan National Laboratory for Optoelectronics and School of Optical and Electronic Information Huazhong University of Science and Technology Wuhan China; ^2^ Optics Valley Laboratory Hubei China; ^3^ Hubei Optical Fundamental Research Center Wuhan China; ^4^ China Electric Power Research Institute Beiiing China

**Keywords:** acoustic hyperbolic polaritons, edge‐confined modes, light–matter interaction, molecular sensing

## Abstract

Hyperbolic polaritons (HPs) supported by van der Waals (vdW) materials enable exceptionally strong light–matter interactions through deep subwavelength confinement. This confinement can be further enhanced when a polaritonic mode couples to its mirror image in a metallic substrate, giving rise to acoustic hyperbolic polaritons (AHPs). While most previous studies have focused on volume‐confined AHPs (v‐AHPs), edge‐confined AHPs (e‐AHPs) remain experimentally elusive. Here, we provide the first near‐field observation of e‐AHPs by launching and imaging them in the prototypical sample of hexagonal boron nitride (hBN) on a gold substrate. Unlike v‐AHPs propagating inside hBN, these e‐AHPs are guided along the hBN edges and exhibit shorter polariton wavelengths, as revealed by both near‐field imaging and extracted dispersion relations. Enhanced vibrational strong coupling in e‐AHPs, experimentally verified by monitoring molecular‐induced near‐field responses, demonstrates their suitability for high‐sensitivity molecule detection. These distinctive properties establish e‐AHPs as a promising platform for optical sensing and on‐chip nanophotonic devices.

## Graphical abstract

This work demonstrates a new type of light wave—edge‐confined acoustic hyperbolic polaritons—in hexagonal boron nitride on gold. These waves travel along crystal edges with nanoscale confinement and strongly interact with molecular vibrations, enabling highly sensitive detection of tiny molecular layers. The findings open pathways toward compact mid‐infrared sensors and integrated nanophotonic devices.

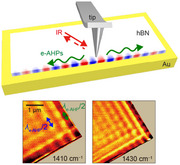

## 1 Introduction

The inherent structural anisotropy of van der Waals (vdW) crystals provides a versatile platform for engineering optical responses far beyond the limits of conventional materials. This anisotropy finds its notable expression in the emergence of hyperbolic polaritons (HPs), which arise in frequency ranges where the principal components of the permittivity tensor carry opposite signs, producing highly anisotropic hyperbolic dispersion and subdiffractional confinement [1, 2, 3, 4, 5, 6, 7, 8]. There are two forms of HPs existing in a vdW flake, depending on their distances from the crystal's sidewalls (the flake edges): volume‐confined mode that appears in the interior of the flake (v‐HPs) [9, 10, 11, 12] and edge‐confined mode that is strongly bound to the edge (e‐HPs) [13]. The former enables ultra‐large in‐plane wavevectors and deep subwavelength guiding, whereas the latter exhibits stronger lateral confinement (larger wavevector) than the former and can route energy around sharp corners of vdW layers. This edge‐bound character originates from boundary‐induced electromagnetic conditions that give rise to high‐momentum surface modes whose fields decay into the interior, suppressing bulk propagation. Together, these features lay the groundwork for diverse nanophotonic applications, including super‐resolution imaging [14, 15], field‐enhanced molecular sensing [16, 17, 18, 19], and subdiffractional waveguiding [20].

To push field confinement further beyond the limits of conventional HPs, the concept of acoustic hyperbolic polaritons (AHPs) has recently been introduced [21]. These modes originate from the near‐field hybridization of a polaritonic excitation in a thin vdW layer with its electromagnetic mirror image in an adjacent metallic substrate [21, 22, 23, 24, 25, 26], in close analogy to acoustic plasmons in graphene/metal heterostructures [27, 28, 29, 30, 31, 32, 33]. As a result of this coupling, AHPs acquire dispersions with reduced group velocity and exhibit markedly stronger vertical and lateral field localization than their conventional HP counterparts [21, 22, 23, 24, 25]. Such extreme confinement opens new avenues for enhancing light–matter interactions at the nanoscale, including vibrational strong coupling, single‐molecule detection, and on‐chip integration. However, most prior studies have focused exclusively on volume‐confined AHPs (v‐AHPs) propagating inside the vdW layer [22, 23, 24, 25]. By contrast, their edge‐confined counterparts (e‐AHPs)—supposed to exhibit even stronger lateral confinement and unique guiding capabilities along crystal boundaries—have been neither theoretically analyzed nor measured in experiments yet. This absence of experimental evidence has left the distinctive properties of e‐AHPs and their potential for extreme optical sensing or energy routing largely untapped.

In this work, we report the first real‐space observation and investigation of e‐AHPs in a prototypical vdW material, hexagonal boron nitride (hBN) on a gold substrate. Using scattering‐type scanning near‐field optical microscopy (s‐SNOM), we directly visualize these modes where they are tightly bound to and guided along crystal edges. Dispersion analysis demonstrates that e‐AHPs exhibit stronger field confinement than their volume‐confined counterparts (v‐AHPs), a result corroborated by numerical simulations. To quantify their ability to amplify light‐matter interactions, we probe the vibrational coupling between AHPs in hBN and an overlayer of 4,4’‐bis(N‐carbazolyl)‐1,1’‐biphenyl (CBP) molecules. Experiments and simulations consistently show a clear enhancement of vibrational coupling for e‐AHPs over v‐AHPs and conventional HPs, evidenced by a larger mode splitting at a fixed molecular thickness. Measurable back‐bending in the dispersion persists even for ultrathin molecular films, highlighting their sensitivity.

## 2 Results and Discussion

### 2.1 Near‐Field Imaging of Edge‐Confined Acoustic Hyperbolic Polaritons

**FIGURE 1:**
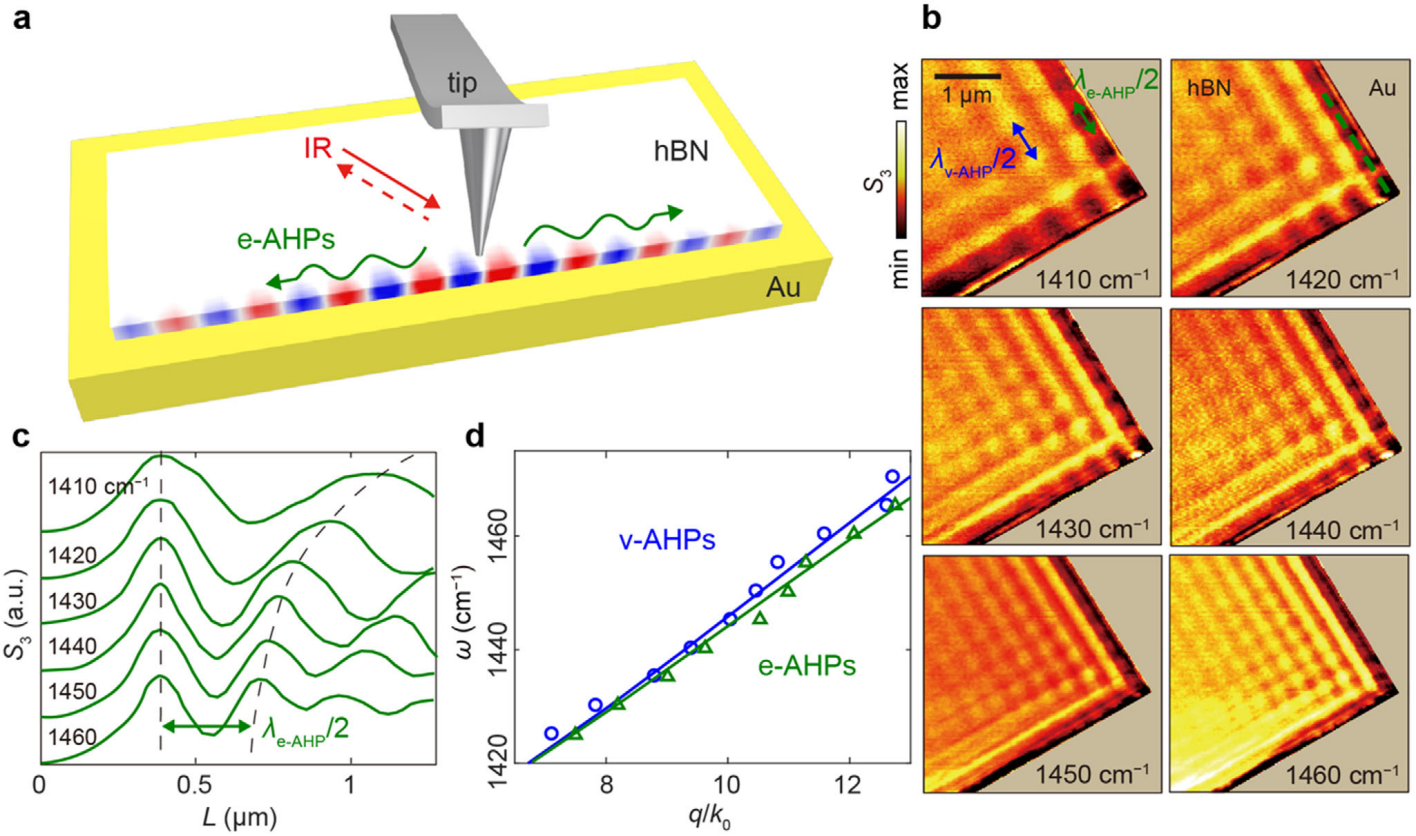
Near‐field imaging of edge‐ and volume‐confined AHPs in hBN flake on Au. (a) Schematic of the experiment overlaid with the simulated electric field distribution of edge‐confined AHPs (e‐AHPs). The mid‐IR illumination excites AHPs, and the s‐SNOM tip detects the near‐field signal. Green arrows indicate the propagation direction of e‐AHPs. (b) s‐SNOM amplitude (*S*_3_) images at selected frequencies. Green and blue double‐headed arrows in the first image (1410 cm^−1^) indicate the periodic near‐field oscillations at the edge and inside the hBN/Au sample, corresponding to the half wavelengths of e‐AHPs and v‐AHPs, respectively. The Au region is colored light beige for enhanced fringe visibility. (c) Normalized line profiles extracted along the green dashed lines in (b). All curves are normalized to their maximum value. (d) Experimental (symbols) and simulated (solid curves) dispersions of e‐AHPs (green) and v‐AHPs (blue), obtained from line profiles like those in (c). *k*_0_ is the free‐space wavevector.

We visualized edge‐confined acoustic hyperbolic polaritons in real space using s‐SNOM (see Methods) [9, 13, 34, 35, 36]. As shown in Figure 1a, the mid‐infrared beam illuminates the metallic tip of s‐SNOM, which launches polaritons at the edge of an hBN flake (thickness, 62 nm) placed on a gold substrate. These excitations propagate away from the tip along the crystal edge (green arrows). The overlaid simulated electric‐field distribution corresponds to the out‐of‐plane electric field component *Ez*, the dominant contributor to the s‐SNOM signal, and illustrates how the polariton field is tightly confined to the boundary, highlighting the edge‐guided nature of e‐AHPs. The s‐SNOM amplitude images (Figure 1b, *S*_3_) recorded at multiple frequencies (1410–1460 cm^−1^) display two distinct sets of interference fringes (see Figure S1 for near‐field images from an additional hBN flake). Inside the hBN flake, fringes parallel to the flake edges are clearly visible which arise from volume‐confined AHPs (v‐AHPs): the tip‐launched and edge‐reflected acoustic polariton waveguide mode together create standing‐wave patterns. The fringe spacing corresponds to half of the v‐AHP wavelength (*λ*_v‐AHP_/2, blue arrow in Figure 1b), in analogy to previous observations for conventional hyperbolic polaritons on dielectric substrates. In addition to these well‐known v‐AHP fringes, we observe a string of alternating bright and dark spots aligned along the flake edge. These edge‐localized oscillations, attributed to e‐AHPs, result from the interference between tip‐launched and corner‐reflected edge‐guided modes. Compared to v‐AHPs, the spacing of these oscillations is reduced (*λ*_e‐AHP_/2, green arrow in Figure 1b), evidencing a shorter polariton wavelength and thus a higher lateral confinement for e‐AHPs. A simulated near‐field image exhibiting the same edge‐guided interference pattern is provided in Figure S2, showing good agreement with the experimental observations.

Despite this stronger confinement, e‐AHPs share several propagation characteristics with their volume‐confined counterparts. For both modes, the amplitude of fringe patterns exhibits a similar decay with increasing distance from the corner, suggesting comparable propagation characteristics in terms of propagation length, quality factor, and lifetime (Figure S3). The overall interference pattern in Figure 1b closely resembles that observed in hBN flakes on SiO_2_/Si substrates, where edge‐ and volume‐confined conventional hyperbolic polaritons (e‐HPs and v‐HPs) are supported [13]. To obtain the dispersion relation of e‐AHPs, we extracted s‐SNOM line profiles at multiple frequencies along the green dashed line in Figure 1b. The normalized profiles (each scaled to its maximum) are plotted in Figure 1c (additional frequencies are provided in Figure S4a). From these profiles, the e‐AHP wavelength *λ*_e‐AHP_ was determined by measuring the distance between adjacent intensity peaks (*λ*_e‐AHP_/2). The corresponding propagation constants are then calculated as *q*_e‐AHP_ = 2π/*λ*_e‐AHP,_ and the experimental dispersion of e‐AHPs is obtained (green triangles in Figure 1d).

For comparison, the experimental dispersion of volume‐confined AHPs (blue circles in Figure 1d) was obtained likewise (see Figure S4b, for v‐AHP line profiles). Both experimental data sets show excellent agreement with the calculated dispersions (green and blue solid lines in Figure 1d, see Methods for details). Throughout the studied frequency range, *q*_e‐AHP_ is larger than *q*_v‐AHP_, confirming that the edge mode is more confined than the volume mode. This trend parallels observations for conventional hyperbolic polaritons in hBN, where *q*_e‐HP_ > *q*_v‐HP_ [13].

### 2.2 Vibrational Strong Coupling Between Conventional HPs and Organic Molecules

**FIGURE 2:**
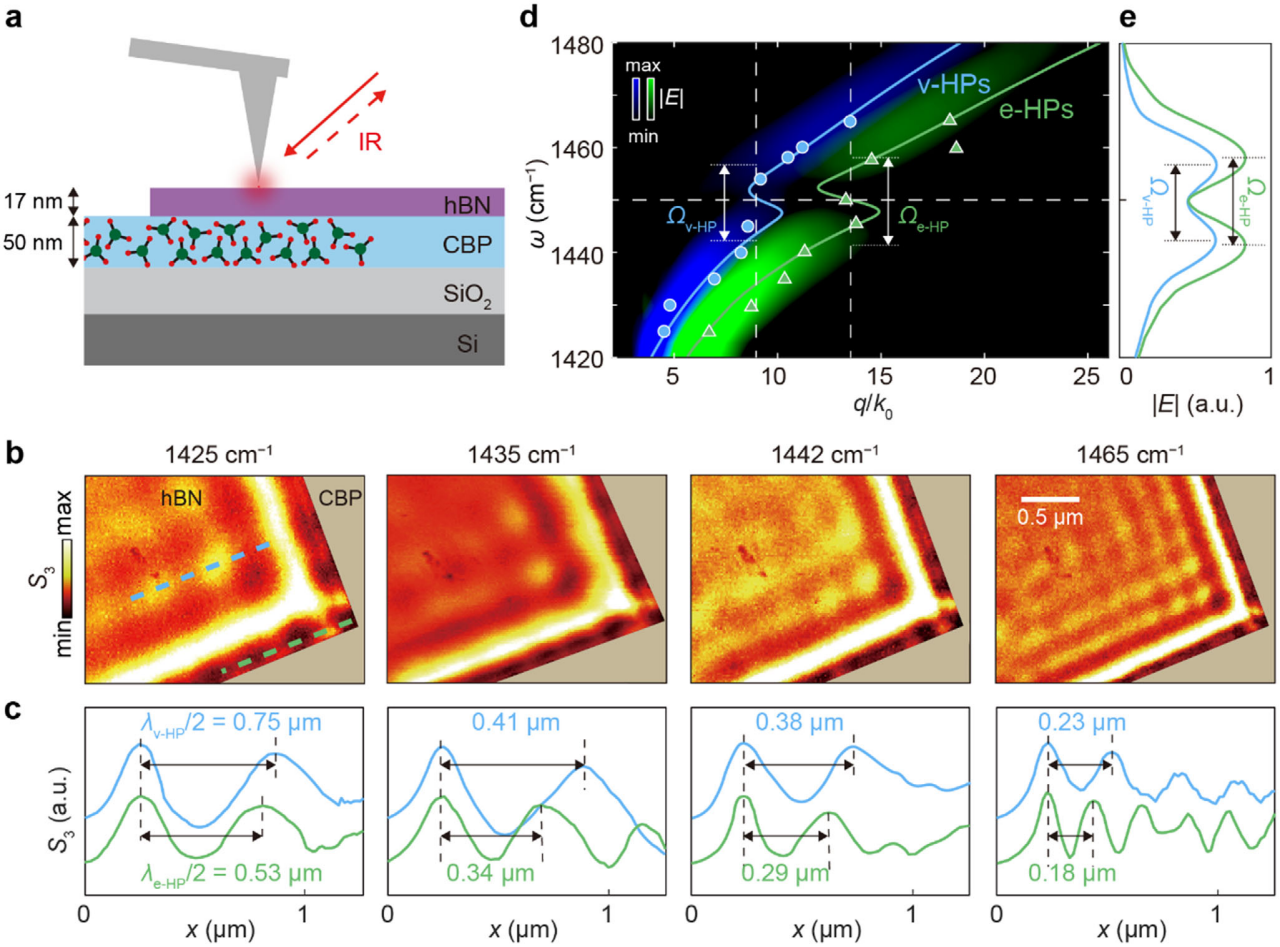
Vibrational strong coupling between conventional HPs in hBN and CBP molecules. (a) Schematic of the experiment. A 50‐nm‐thick CBP layer is placed between a 17‐nm‐thick hBN flake and a SiO_2_ (285 nm)/Si substrate. (b) s‐SNOM amplitude (*S*_3_) images at selected frequencies. (c) Normalized line profiles of e‐HPs (v‐HPs) extracted along the light green (blue) dashed lines in (b). All curves are normalized to their maximum value. (d) Experimental (symbols) and simulated (solid curves and false‐color images) dispersions of edge‐confined HPs (e‐HPs, light green) and volume‐confined HPs (v‐HPs, light blue). Solid curves correspond to calculated dispersions using complex‐valued momenta *q* + *ik*, and false‐color images show Fourier‐transformed amplitudes of simulated line profiles. (e) Line profiles extracted along the two vertical dashed lines in (d).

To investigate the vibrational strong coupling between conventional HPs in hBN and organic molecules, we prepared a layered sample by placing a 50‐nm‐thick film of CBP molecules between an hBN flake (17 nm) and a SiO_2_/Si substrate. Using s‐SNOM, we imaged the sample at multiple frequencies near the CBP vibrational resonance at *ω* = 1450 cm^−1^ (Figure 2b, additional frequencies are shown in Figure S5). Line profiles of edge‐ and volume‐confined HPs (Figure 2c) were extracted from these images along the light green (e‐HPs) and light blue (v‐HPs) dashed lines, respectively. In Figure 2d, the experimental data points (circles and triangles) were obtained by measuring the fringe spacings in the line profiles and converting the corresponding wavelengths to wavevectors. These experimentally derived dispersions show excellent agreement with the calculated dispersions (solid curves), which were derived using complex‐valued wavevectors *q* + *iκ* and real‐valued frequencies *ω*. Importantly, molecular absorption at the CBP vibrational resonance (dashed horizontal line) produces a back‐bending feature in both dispersion curves, marked with vertical double‐headed arrows [17]. This back‐bending is in sharp contrast with the case when CBP molecules are not present, rendering itself as a hallmark of strong light–matter coupling between the molecular vibrations and HPs.

We further verified this strong coupling using an alternative simulation approach: simulated line profiles of e‐HPs and v‐HPs at multiple frequencies were Fourier‐transformed to construct the false‐color (*q*, *ω*) plot in Figure 2d (see Methods). Here, the false‐color plot for e‐HPs (green) and v‐HPs (blue) were combined by assigning them to separate RGB channels and overlaying them in a single composite map for direct comparison. These maps reveal clear anti‐crossing behavior at the CBP resonance for both e‐HPs and v‐HPs. From these anti‐crossings, we extracted mode splittings of the two modes *Ω*_e‐HP_ = 17.4 cm^−1^ and *Ω*_v‐HP_ = 15.1 cm^−1^ (Figure 2e, line profiles along white dashed verticals in Figure 2d). Considering the CBP linewidth (*Γ*_CBP_ = 6.5 cm^−1^) and the uncoupled polariton linewidths (*Γ*_e‐HP_ = *Γ*_v‐HP_ = 1.8 cm^−1^, see Methods), the strong‐coupling condition *C* ≡ Ω^2^/(*Γ*_CBP_^2^/2+*Γ*_HP_^2^/2) > 1 is well satisfied for both e‐HPs (*C* = 13.3) and v‐HPs (*C* = 10.0) [17], underscoring pronounced vibrational strong coupling.

### 2.3 Vibrational Strong Coupling between AHPs and Organic Molecules

**FIGURE 3:**
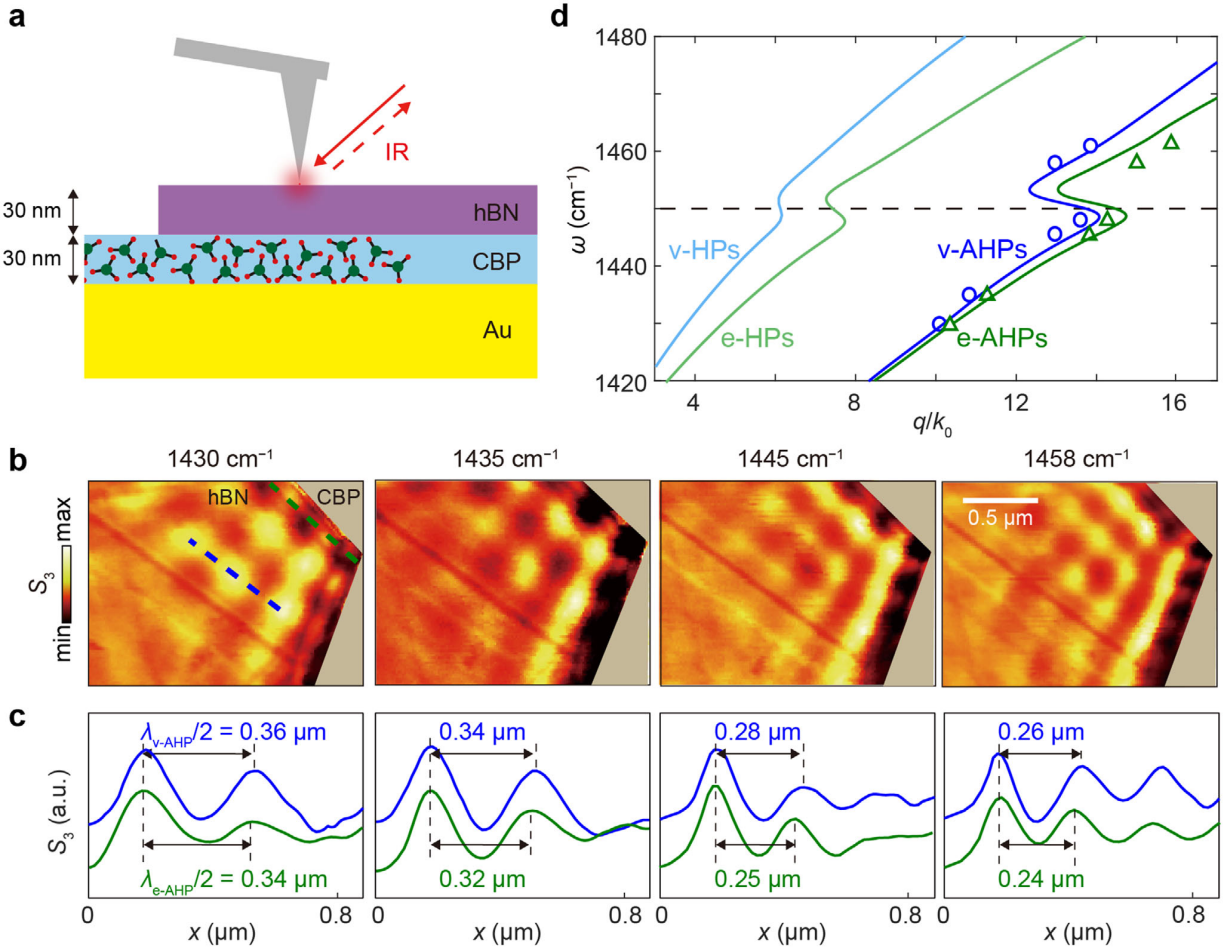
Vibrational strong coupling between AHPs and CBP molecules. (a) Schematic of the experiment. A 30‐nm‐thick CBP layer is placed between a 30‐nm‐thick hBN flake and an Au substrate. (b) s‐SNOM amplitude (*S*_3_) images at selected frequencies. (c) Normalized line profiles of e‐AHPs (v‐AHPs) extracted along the green (blue) dashed lines in (b). All curves are normalized to their maximum value. (d) Experimental (symbols) and simulated (solid curves) dispersions of edge‐confined AHPs (e‐AHPs, green) and volume‐confined AHPs (v‐AHPs, blue), calculated using complex‐valued momenta *q* + *ik*. Calculated dispersions of e‐HPs (light green) and v‐HPs (light blue) on a similar sample with Au replaced by SiO_2_/Si are added for comparison.

Having established vibrational strong coupling for conventional HPs, we extended our study to AHPs to assess their potential for even stronger light–matter interactions with organic molecules. As illustrated in Figure 3a, the SiO_2_/Si substrate was replaced with gold, and both the CBP and hBN layers were 30 nm thick. Following the procedure used in Figure 2, we imaged edge‐ and volume‐confined AHPs at frequencies near the CBP vibrational resonance (Figure 3b, with additional frequencies in Figure S6). Line profiles were extracted from the s‐SNOM images along the green and blue dashed lines, see Figure 3c. To quantify these trends, from measured line profiles we converted the wavelengths to propagation constants, and plotted the resulting dispersions in Figure 3d. The experimental dispersions (symbols) show excellent agreement with numerical simulations (solid curves) based on complex‐valued wavevectors *q* + *iκ* and real‐valued frequencies *ω*. Notably, unlike the case without CBP shown in Figure 1d, both e‐AHPs (green) and v‐AHPs (blue) exhibit back‐bending near the molecular resonance, analogous to the behavior of e‐HPs and v‐HPs in Figure 2b.

To further evaluate the coupling strength, we calculated the amplitudes of the Fourier transforms of simulated line profiles (analogous to those in Figure 3c) and merged the resulting maps to highlight anti‐crossing features at the CBP resonance (Figure S7). From these anti‐crossings, we extracted mode splittings of *Ω*_e‐AHP_ = 14.2 cm^−1^ and *Ω*_v‐AHP_ = 13.8 cm^−1^. Considering the uncoupled acoustic‐mode polariton linewidths (*Γ*_e‐AHP_ = *Γ*_v‐AHP_ = 1.8 cm^−1^), the strong‐coupling criterion *C* > 1 is clearly fulfilled for both e‐AHPs (*C* = 8.9) and v‐AHPs (*C* = 8.4), demonstrating pronounced vibrational light–matter coupling at the edges and within the bulk of the hBN/Au system.

For quantitative comparison, we also calculated the dispersions of edge‐ and volume‐confined HPs supported by the same hBN crystal but the gold substrate was replaced by SiO_2_ (285 nm)/Si. As shown in Figure 3d, compared with these conventional HPs (light green and light blue), the dispersions of AHPs (green and blue) display significantly larger *q* values. Moreover, the extracted mode splittings follow the order *Ω*_e‐AHP_ > *Ω*_v‐AHP_ > *Ω*_e‐HP_ > *Ω*_v‐HP_ (see Figure S8), leading to a corresponding trend in coupling strengths *C*_e‐AHP_ > *C*_v‐AHP_ > *C*_e‐HP_ > *C*_v‐HP_. This hierarchy indicates that AHPs, particularly edge‐confiend AHPs, achieve the strongest light–matter coupling. The superior performance of AHPs originates from their stronger mirror effect at the hBN/metal interface. For e‐AHPs, the field maximum is pushed closer to the interface and exhibits pronounced vertical confinement, dramatically increasing the overlap between the polaritonic field and the CBP layer. Additionally, the boundary‐guided nature of e‐AHPs enhances their in‐plane momentum, resulting in tighter lateral field localization than v‐AHPs and conventional HPs. This combined vertical and lateral confinement amplifies the local field intensity and interaction strength, enabling e‐AHPs to achieve the largest mode splitting and the strongest vibrational coupling among these polariton types, consistent with the mirror‐charge mechanism illustrated in Figure S9.

### 2.4 Thickness‐Dependent Coupling between AHPs and Thin Organic Molecular Layers

**FIGURE 4:**
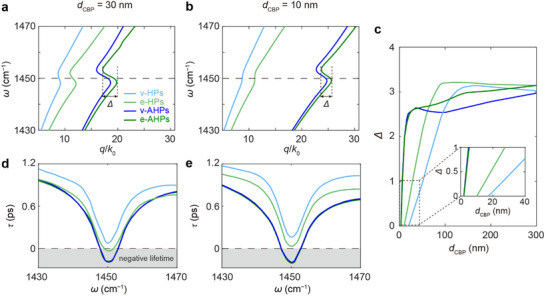
Dispersions of HPs and AHPs with varied thickness of CBP molecules. (a,b) Calculated dispersions of v‐HPs (light blue), e‐HPs (light green), v‐AHPs (blue), and e‐AHPs (green) for a 20‐nm‐thick hBN layer on CBP film of thickness *d*_CBP_ = 30 nm (a) and *d*_CBP_ = 10 nm (b). The back‐bending magnitude *Δ* is defined as the largest horizontal separation of the dispersion curve near the molecular resonance (horizontal dashed line). (c) Back‐bending magnitude *Δ* as a function of the CBP thickness. Inset: Enlarged view of selected region. (d,e) Corresponding lifetimes of all four modes for *d*_CBP_ = 30 nm (d) and *d*_CBP_ = 10 nm (e).

To assess how the mirror‐mode field confinement of AHPs improves coupling efficiency with ultrathin organic layers, we numerically calculated the dispersions and lifetimes of AHPs and conventional HPs for hBN on organic layers of different thicknesses. Figure 4a,b display the calculated dispersions of v‐HPs (light blue), e‐HPs (light green), v‐AHPs (blue), and e‐AHPs (green) for two representative CBP thicknesses, *d*_CBP_ = 30 and 10 nm. These dispersions exhibit similar features to those in Figure 3d, where both hBN and CBP layers are 30 nm thick. As *d*_CBP_ decreases from 30 to 10 nm, the dispersions of v‐AHPs and e‐AHPs laterally shift to larger wavevectors, while the dispersions of v‐HPs and e‐HPs do not significantly change except the weakened back‐bending. This shows that, in contrast to HPs, the field confinement ratio of AHPs can further increase as the CBP thickness reduces.

The quantitative dependence of back‐bending magnitude *Δ* on CBP thickness *d*_CBP_ is summarized in Figure 4c. Here we define *Δ* as the largest horizontal separation of the dispersion curve near the molecular vibrational resonance (horizontal dashed line). When the CBP thickness is small, there is a linear correlation between organic layer thickness and back‐bending magnitude *Δ* for all studied modes. For AHPs (green and blue), saturation of *Δ* occurs at smaller *d*_CBP_ (> 30 nm) than for conventional HPs (light green and light blue, > 100 nm). More importantly, at CBP thickness below ∼10 nm (see inset), finite back‐bending persists for AHPs but vanishes for conventional HPs. This heightened sensitivity originates from the mirror effect of AHPs, whose tightly confined fields maintain efficient coupling with ultrathin CBP layers.

A further comparison of polariton lifetimes shows that the AHPs does not suffer a significant lifetime reduction due to the metallic substrate. The polariton lifetimes *τ* were extracted according to *τ* = *L*/*v*_g_, where the propagation length is *L* = 1/*κ* and the group velocity *v*_g_ = d*ω*/d*q*.

As shown in Figure 4d,e, with *d*_CBP_ = 30 nm or 10 nm, the lifetimes remain in the range of 0.8–1.1 ps away from the molecular resonance at 1450 cm^−1^. Near resonance, however, the lifetimes drop sharply, and for e‐AHPs and v‐AHPs even become negative due to negative group velocity, a behavior indicating strong coupling between hyperbolic polaritons and molecular vibrations according to previous studies [17]. We note that the negative lifetime arises from back‐bending of the dispersion under strong coupling, and does not represent physical gain or true negative decay. Overall, our calculations highlight the potential of AHPs for achieving strong coupling with ultrathin molecular layers, offering both a substantially lower detection threshold and well‐preserved polariton lifetimes.

## 3 Conclusion

In summary, we directly mapped edge‐confined AHPs at the edges of hBN flakes on gold. These modes are tightly bound to crystal boundaries, guided along them, and display shorter wavelengths than their volume‐confined counterparts, as verified by near‐field imaging and dispersion analysis. The gold‐induced mirror effect, combined with stronger field confinement, markedly enhances their vibrational strong coupling with thin organic layers, enabling the detection of very small molecular amounts without the need for complex nanostructuring. Beyond ultrasensitive molecular sensing, the exceptional confinement and edge‐guided nature of AHPs open promising opportunities for compact on‐chip mid‐infrared spectroscopy, tunable nanophotonic components, and edge‐specific studies of light–matter interactions in a broad range of van der Waals materials.

## 4 Methods

### 4.1 Sample Preparations

Two SiO_2_/Si substrates were prepared, one of which was coated with a Ti (5 nm)/Au (300 nm) layer deposited directly onto the surface. Both the bare SiO_2_/Si substrate and the Au‐coated substrate were then covered with CBP molecular layers deposited by thermal evaporation. Hexagonal boron nitride (hBN) flakes were mechanically exfoliated from bulk crystals and subsequently dry‐transferred onto the samples.

### 4.2 s‐SNOM Experiments

Near‐field imaging was performed using a commercial scattering‐type scanning near‐field optical microscope (s‐SNOM, Neaspec GmbH) based on a tapping‐mode atomic force microscope (AFM). A wavelength‐tunable continuous‐wave quantum cascade laser (QCL) provided *p*‐polarized mid‐infrared illumination focused onto a metal‐coated (Pt/Ir) AFM tip. The tip was oscillated at a frequency of ∼270 kHz with an amplitude of ∼60 nm. The backscattered light was detected via a pseudoheterodyne interferometer. To suppress background scattering from the tip shaft and sample, the detector signal (*S*_3_) was demodulated at the third harmonic.

### 4.3 Numerical Simulations

The electric field distributions and dispersion relations discussed in this work were numerically simulated via finite element analysis using COMSOL Multiphysics. The dielectric function of gold was adopted from Ref. [37]. For hexagonal boron nitride, the whole isotopically enriched h^10^BN flake was modeled as a homogeneous anisotropic slab using a Lorentz oscillator model for both in‐plane (⊥) and out‐of‐plane (∥) permittivity components:

$$\varepsilon_{\mathrm{hBN},m}=\varepsilon_{\infty ,m}\;\left(1+\frac{\omega_{\mathrm{LO},m}^{2}-\omega_{\mathrm{TO},m}^{2}}{\omega_{\mathrm{TO},m}^{2}-\omega^{2}-i\omega \Gamma_{m}}\right)$$

where the subscript *m* denotes the direction of oscillator components (⊥ or ∥), *ε*_∞_ is the high‐frequency permittivity, *ω*_TO_ and *ω*_LO_ are the transverse and longitudinal optical phonon frequencies, *Γ* is the phonon damping rate. The parameters were set following Ref. [38]: *ε*_∞,⊥_ = 5.1, *ω*_TO,⊥_ = 1394.5 cm^−1^, *ω*_LO,⊥_ = 1650 cm^−1^, *Γ*_⊥_ = 1.8 cm^−1^, *ε*_∞,∥_ = 2.5, *ω*_TO,∥_ = 785 cm^−1^, *ω*_LO,∥_ = 845 cm^−1^, *Γ*_∥_ = 1 cm^−1^. The dielectric function of CBP was modeled as *ε*_CBP_ = *ε*_CBP,∞_  + *ε*_CBP,vib_, where *ε*_CBP,∞_ = 2.8 is the non‐dispersive background and *ε*_CBP,vib_ represents the vibrational contributions described by four Lorentz oscillators

$$\varepsilon_{\mathrm{CBP},\mathrm{vib}}=\sum_{k=1}^{4}\frac{S_{k}^{2}}{\omega_{k}^{2}-\omega^{2}-i\omega \gamma_{k}}$$

with oscillator parameters (*S_k_*, $\omega_{k}^{2}$ and *γ*_*k*_ represent the intensity, central frequency, and damping of the *k*‐oscillator): *ω*_1_ = 1450 cm^−1^, *γ*_1_ = 6.4 cm^−1^, *S*_1_ = 128 cm^−1^, *ω*_2_ = 1478.6 cm^−1^, *γ*_2_ = 4.4 cm^−1^, *S*_2_ = 47 cm^−1^, *ω*_3_ = 1500.1 cm^−1^, *γ*_3_ = 9.4 cm^−1^, *S*_3_ = 91 cm^−1^, *ω*_4_ = 1507.4 cm^−1^, *γ*_4_ = 6.1 cm^−1^, *S*_4_ = 99 cm^−1^ [17].

To obtain the polariton dispersion, the mode solver was used to compute the complex wavevector of the guided modes in hBN layer on Au. Perfectly matched layers were applied to suppress artificial reflections, and mesh refinement was concentrated in the hBN and edge regions to ensure accurate evaluation of field confinement and decay profiles. Near‐field images were simulated by placing a vertically oriented dipole source above the hBN surface and computing the out‐of‐plane electric field component Re(*E_z_*) at the dipole position while scanning across the structure, reproducing the spatial fringe patterns observed in s‐SNOM experiments.

Anti‐crossing behavior of polaritons was analyzed using Lumerical FDTD simulations. A broadband pulsed plane wave (wavelength range: 6–8 µm) was incident normally on the sample surface, with polarization along the *x*‐direction. The spatial mesh was set to Δ*x* = 0.03 µm, Δ*y* = 0.01 µm, and Δ*z* = 0.03 µm to resolve near‐field features. Perfectly matched layer boundary conditions were applied in all directions. Frequency‐domain power monitors were placed along the hBN edge (for edge‐confined HPs) and in the interior region (for volume‐confined HPs), each spanning 6 µm along the *x*‐direction. The recorded field profiles were Fourier‐transformed along the *x*‐direction to obtain momentum‐space dispersion maps (*k_x_*, *ω*), as shown in Figure 2d.

### 4.4 Calculation of Uncoupled Polariton Linewidth

The uncoupled polariton linewidth *Γ* was derived from a reference hBN/dielectric/SiO_2_/Si structure, where the CBP layer was replaced by a dielectric with *ε*  = *ε*_CBP,∞_ **. Dispersion relations were computed analytically using a transfer matrix method with complex frequencies *ω* − *iγ* and real wavevectors *q*. The imaginary part of *γ* was extracted, and the uncoupled polariton linewidth was defined as *Γ* = 2*γ* [17].

## Conflicts of Interest

The authors declare no conflicts of interest.

## Supporting information




**Supporting File**: advs73539‐sup‐0001‐SuppMat.pdf.

## Data Availability

The data that support the findings of this study are available from the corresponding author upon reasonable request.
