# Supplementary material for: Observation of Edge‐Confined Acoustic Hyperbolic Polaritons in van der Waals Materials
Source: Adv Sci (Weinh). 2026 Jan 4;13(14):e20556. doi: 10.1002/advs.202520556 (PMC12970239; doi:10.1002/advs.202520556)
Supplement: Supplementary file 1 — Supporting File: advs73539‐sup‐0001‐SuppMat.pdf. [file ADVS-13-e20556-s001.pdf]

Supporting Information

Observation of edge-confined acoustic hyperbolic polaritons in van der Waals materials

Tianning Zhang[1,2,3,4‡], Xiaojie Jiang[1,2,3‡], Xiaosheng Yang[1,2,3], Xinliang Zhang[1,2,3], Peining Li[1,2,3]**

1. Wuhan National Laboratory for Optoelectronics and School of Optical and Electronic Information, Huazhong University of Science and Technology, Wuhan 430074, China
2. Optics Valley Laboratory, Hubei 430074, China
3. Hubei Optical Fundamental Research Center, Wuhan 430074, China
4. China Electric Power Research Institute, Beiiing 102209, China

*Corresponding authors: yang_xs@hust.edu.cn, lipn@hust.edu.cn
‡These authors contributed equally

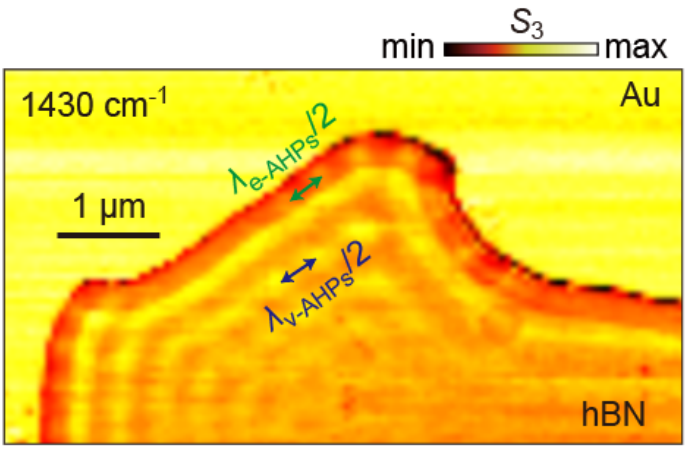

Figure S1. Near-field imaging of a 59-nm-thick hBN flake on gold substrate.

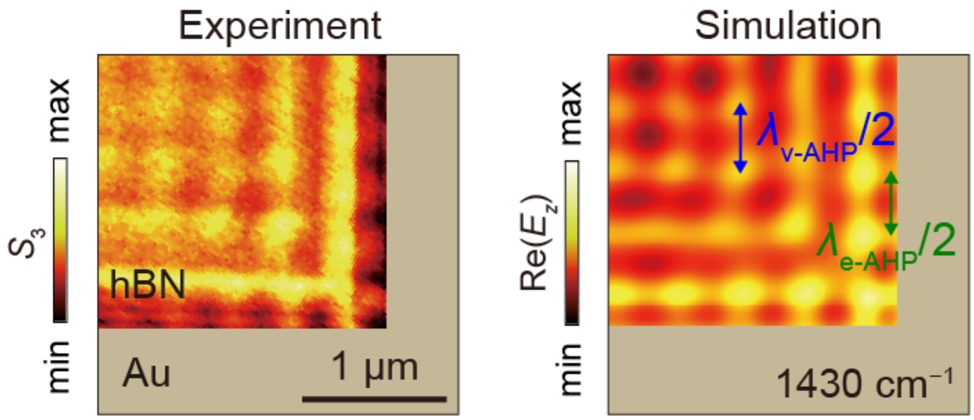

Figure S2. Experimental and simulated near-field image of hBN on Au at 1430 cm^{-1}. The experimental image is the same as taken from Figure 1b of the main text.

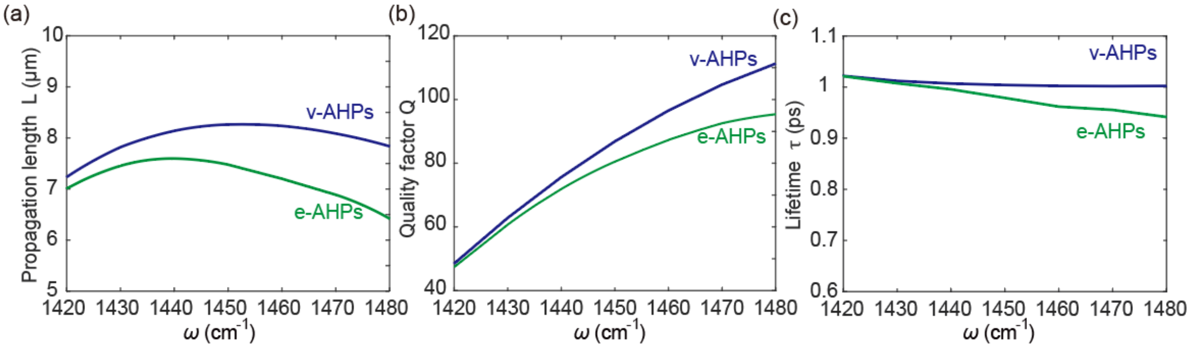

Figure S3. Comparison of propagation length, quality factor, and lifetime of e-AHPs and v-AHPs. All quantities were extracted from the complex wavevector $k = q + i\kappa$ obtained using the COMSOL mode solver, where the propagation length is L=1/(2κ), the quality factor Q=q/κ, and the lifetime τ=L/v_g (v_g denotes the group velocity).

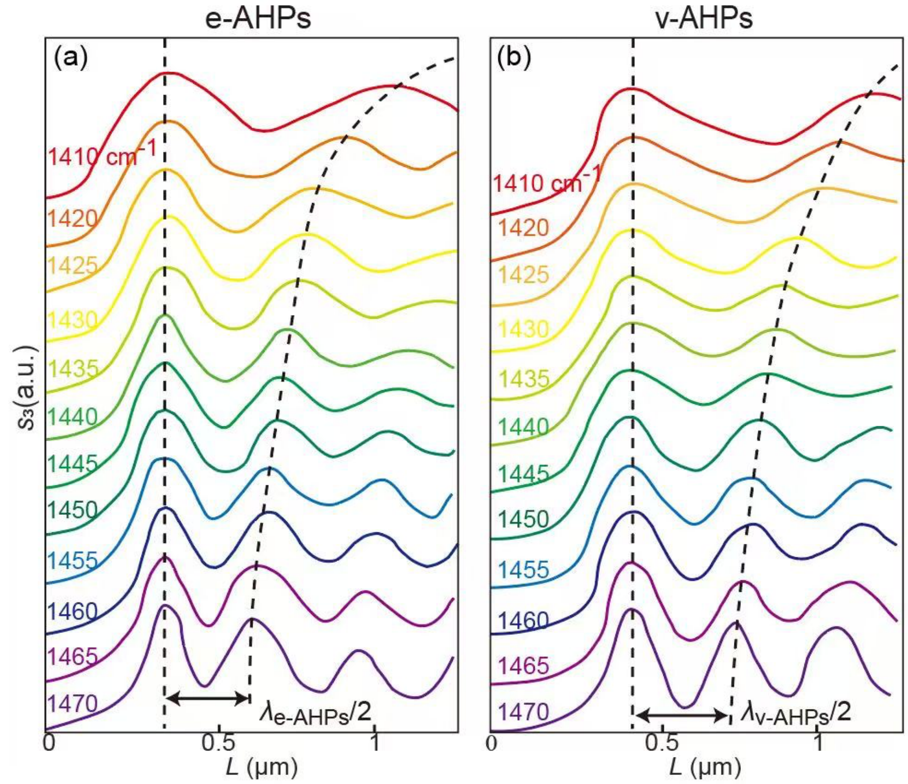

Figure S4. Normalized line profiles of e-AHPs and v-AHPs extracted from s-SNOM images at different frequencies. All curves are normalized to their maximum value.

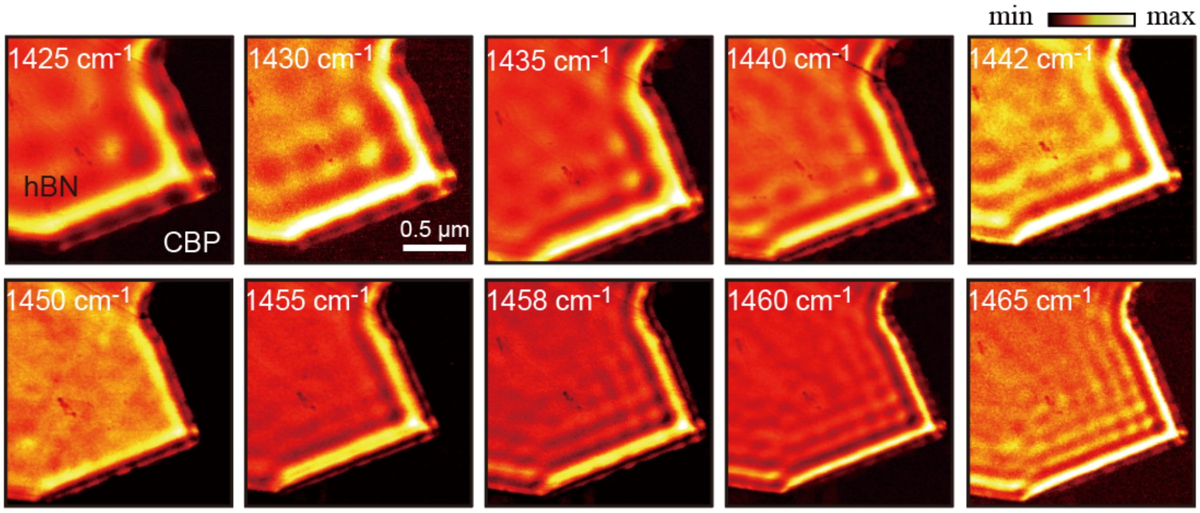

Figure S5. s-SNOM images (amplitude signal S_3) of e-HPs and v-HPs on an hBN/CBP/SiO_2/Si sample at different frequencies.

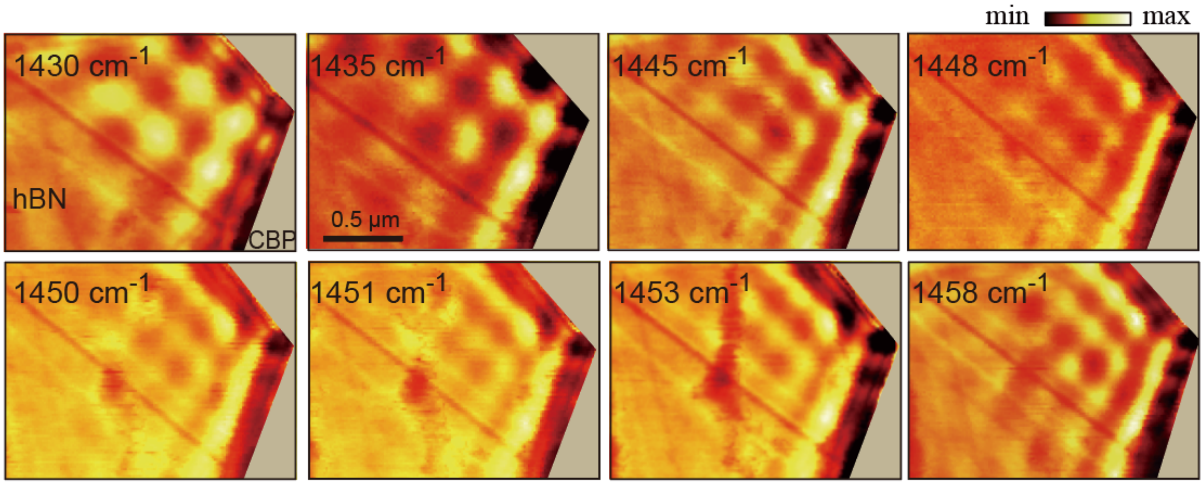

Figure S6. s-SNOM images (amplitude signal S_3) of e-AHPs and v-AHPs on an hBN/CBP/Au sample at different frequencies. To enhance fringe visibility, the CBP region is colored light beige.

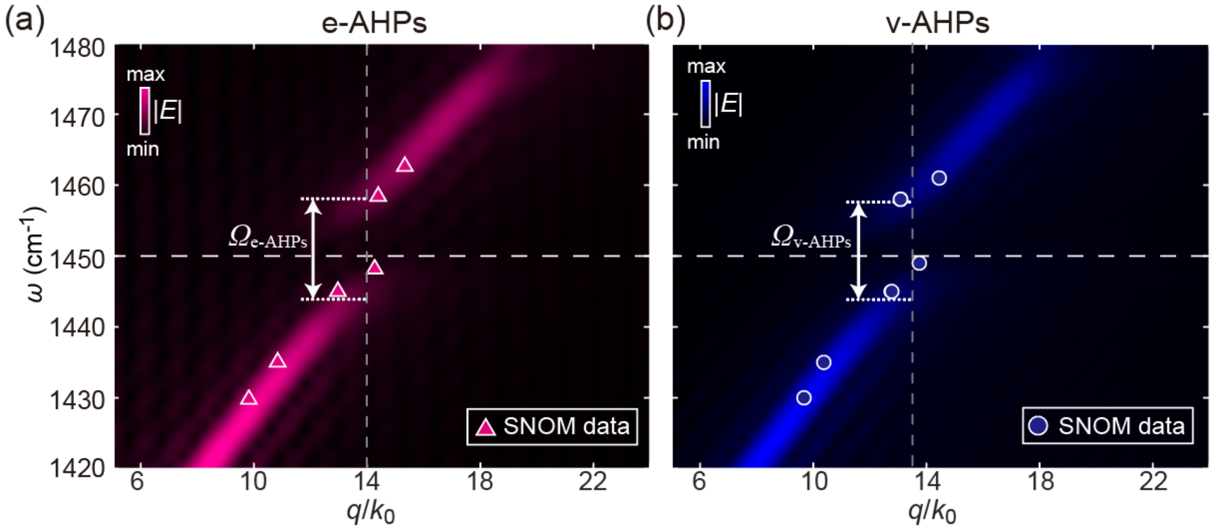

Figure S7. Dispersions of e-AHPs and v-AHPs, obtained by experiments (symbols) and numerical simulations (false color images). The false color images indicate the calculated amplitudes of the Fourier transform of the simulated line profiles such as the ones shown in Figure 3c of main text.

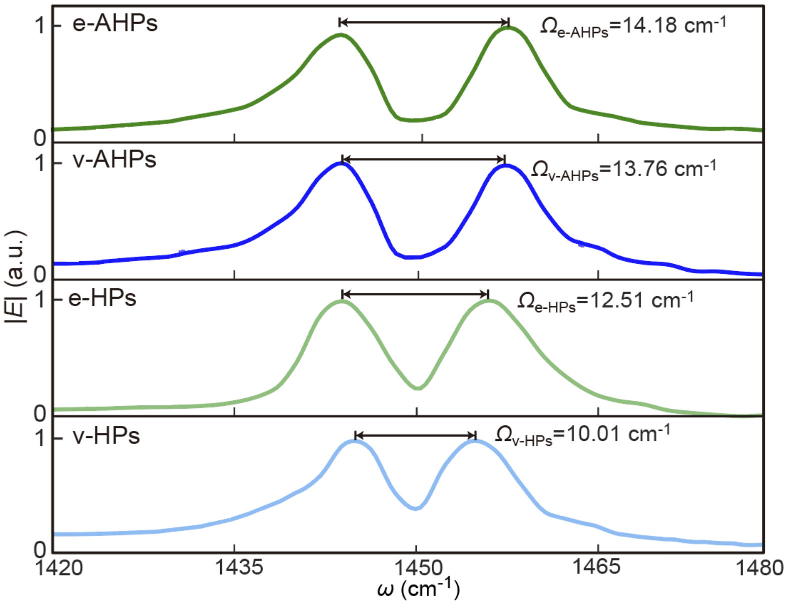

Figure S8. Comparison of mode splitting *Ω* in e-AHPs, v-AHPs, e-HPs, and v-HPs extracted from simulations.

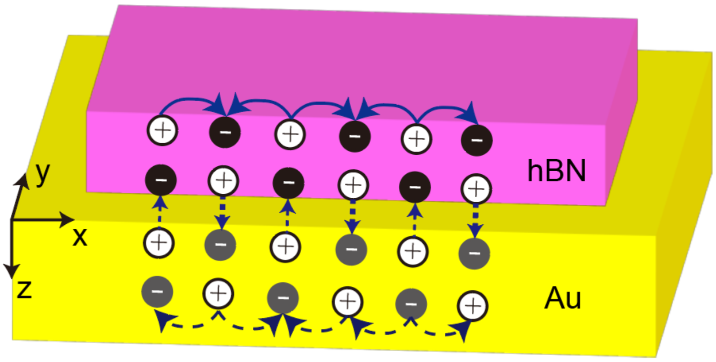

Figure S9. Schematic illustration of mirror-charge formation at the hBN/Au interface and its impact on vibrational coupling. Oscillating charges in the hBN layer induce mirror charges in the metallic substrate due to the large negative permittivity of Au in the mid-infrared. This mirror-charge response enforces an antisymmetric field configuration in the vertical direction, suppressing even-order modes and allowing only odd-order solutions. The resulting hybridization between real and mirror charge distributions compresses the electromagnetic field into the nanoscale region between hBN and Au, substantially enhancing vertical confinement and local field intensity. For e-AHPs, this compressed field profile is further amplified by boundary-guided lateral confinement, increasing both vertical overlap and in-plane momentum. Consequently, the interaction strength with molecular vibrations is significantly enhanced, enabling strong coupling even for ultrathin molecular layers.
